# Network pharmacology and AI in cancer research uncovering biomarkers and therapeutic targets for RALGDS mutations

**DOI:** 10.1038/s41598-025-91568-x

**Published:** 2025-03-29

**Authors:** S. Mohammed Zaidh, Hariharan Thirumalai Vengateswaran, Mohammad Habeeb, Kiran Balasaheb Aher, Girija Balasaheb Bhavar, N. Irfan, K N V Chenchu Lakshmi

**Affiliations:** 1 Crescent School of Pharmacy, BS Abdur Rahman Crescent Institute of Science and Technology, Chennai, 600048 India; 2 D3 Drug Tech Lab Pvt Ltd, Chennai, 600048 India; 3 Department of Pharmaceutical Quality Assurance, Shri Vile Parle Kelavani Mandal’s Institute of Pharmacy, Dhule, Maharashtra 424001 India; 4 Department of Pharmaceutical Chemistry, Shri Vile Parle Kelavani Mandal’s Institute of Pharmacy, Dhule, Maharashtra 424001 India; 5 Department of Pharmaceutical Chemistry, KL College of Pharmacy, Koneru Lakshmaiah Education Foundation, Green Fields, Vaddeswaram, A.P 522302 India

**Keywords:** KRAS–RALGDS, Genomics statistics, Pathway analysis, Multi-omics, Molecular modelling, Cancer, Computational biology and bioinformatics, Drug discovery

## Abstract

The lack of target therapies is accountable for the higher mortality of various types of cancer. To address this issue, we selected a target mutated Kirsten rat sarcoma virus oncogene homologue, which plays a significant role in various cancers. Our study aims to identify selective biomarkers and develop diagnostic and therapeutic strategies for KRAS-associated genes using artificial intelligence. Initially, Genomic data, cancer epidemiology, proteomics network interactions, and omics enrichment were analyzed. Structured E-pharmacophore model aided in capturing the binding cavity using eraser algorithms and fabricating a new selective lead compound for the KRSA. The selective molecule was abridged inside the binding cavity and stability was validated through 100 ns molecular dynamics simulations. Epidemiological-neural network studies indicated KRAS mutations leads 40 types of cancer, exclusively pancreatic and colorectal cancers, with diploid and missense mutations as primary factors. Pathway analysis highlighted the involvement of the MAPK and RAS signaling pathways in cancer development and proteomics analysis identified RALGDS as a key protein. Protein-based pharmacophore analysis mapped the biologically active features such as donor, acceptor and aromatic ring with the designed ligands. The results of interaction interpretation illustrate that the amino acid Tyr566 formed an H-bond interaction with the amine group of the octyl ring system and 20 amino acids crafted to properly orient the molecule to fit inside the polar cavity of KRAS protein. The MMGBSA score of − 53.33 kcal/mol conformed to the well-configured binding with KRSA and realistic model simulation exposed the π–π, π–cationic and hydrophobic interactions stabilised the molecule inside the KRSA protein throughout 100 ns simulation. The study demonstrates the vitality of AI and network pharmacology to identify potential-target biomarkers for KRAS-associated genes, paving the way for improved cancer diagnostics and therapeutics.

**Supplementary Information:**

The online version contains supplementary material available at 10.1038/s41598-025-91568-x.

## Introduction

The most often mutated oncogene (Kirsten Rat Sarcoma Viral Oncogene Homologue (KRAS)) is responsible for a diversity of malignancies, such as NSCLC-non-small-cell lung cancer^1^, PDAC-pancreatic ductal adenocarcinoma, & CRC-colorectal cancer^2–6^. The discovery of certain inhibitors and the recognition of driver genes have completely changed the way that cancer is treated, greatly increasing progression-free survival and lowering toxicity in targeted therapy^7–10^. One of the extremely aggressive and severe diseases is pancreatic cancer which has a poor prognosis and limited available treatments. Mutations in the KRAS oncogene are a key factor among the many genetic changes linked to the onset and spread of pancreatic cancer. Roughly 90% of individuals with pancreatic cancer have KRAS mutations, which are among the most frequent genetic abnormalities discovered in these patients^11–14^. Targeted therapies for other mutations, like EGFR and ALK gene fusion, have advanced; however, KRAS has proven to be a difficult disease to treat, with few effective treatments available until sotorasib for the KRAS (G12C) subtype was recently approved^15^. Because of its inherent qualities, targeting KRAS has proven to be particularly difficult in contrast to other oncogenes. There has been limited success or selectivity in several indirect ways, such as focusing on downstream signalling effectors, using epigenetic techniques, and employing synthetic lethality tactics. Furthermore, KRAS mutant patients frequently show poor response to conventional therapy, resulting in the need for more research in this field^16–19^. The development of inhibitors that selectively target the KRAS oncogene presents a viable path in the search for targeted treatments for pancreatic cancer. However, considering the complexity and dynamic nature of KRAS signalling, this has proven to be a difficult undertaking^20^. According to research by Anabela Ferreira et al., and team, the RAL guanine nucleotide dissociation stimulator (RALGDS) functions as a key downstream effector in KRAS signaling, acting as a GTP/GDP exchange factor to promote the GDP-GTP conversion for RAS-like (RAL) proteins. This activation of RAL GTPases by RALGDS contributes significantly to pro-survival mechanisms, supporting cellular proliferation and cell cycle progression. Recent studies highlight RALGDS’s essential role in modulating KRAS-driven oncogenic pathways, linking RALGDS activity to enhanced tumor growth and metastasis in various cancers. RALGDS thus emerges as a critical mediator in KRAS signaling, with potential therapeutic targeting implications. Continued research focuses on the mechanistic pathways and regulatory interactions of RALGDS within the KRAS network to uncover novel intervention strategies. An essential modulator of the KRAS pathway, RALGDS (RAS-specific guanine nucleotide exchange factor) has drawn interest as a possible zone for the fabrication of novel molecules to treat pancreatic cancer. An important development in the ongoing therapy of pancreatic cancer is the discovery of RALGDS-targeted inhibitors. By selectively targeting RALGDS, these inhibitors aim to interfere with the KRAS signalling cascade, which may impede the growth and viability of cancer cells that carry KRAS mutations^21^. These inhibitors offer hope for more potent and focused therapy for pancreatic cancer patients, potentially enhancing their prognosis and quality of life, even if research and clinical application of these drugs are still in their early stages^22^. The discovery of oncogenes linked to rodent sarcoma virus genes, including Kirsten Rat Sarcoma Virus and Murine Sarcoma Virus, began the quest towards understanding KRAS. These oncogenes are homologous to human KRAS, which has been critical in starting a series of signalling molecules that affect cell proliferation, differentiation, chemotaxis, and apoptosis. We shall^23^ explore the complex world of KRAS as we go along, including its protein, gene, carcinogenic function, and the exciting opportunities it offers for cancer treatment^24,25^.

To find potential therapeutic targets and the underlying molecular mechanisms influenced by the inhibitors, gene enrichment analysis for K-Ras-associated RALGDS targeted inhibitors involves gathering gene expression data from altered and unaltered gene tissues, selecting gene sets associated with K-Ras and RALGDS pathways, preprocessing the data for quality, statistically comparing gene expression changes like mutation, amplification etc.^26^, visualizing the results, interpreting the physiological importance of these changes, and validating the findings. STRING is a database of anticipated and known protein-protein interactions^27^. They involve direct (physical) & indirect (functional) correlations^28^. The process of docking K-Ras gene-associated RALGDS inhibitor pancreatic cancer-targeted drugs involves predicting the binding affinity, finding of allosteric site of the protein by the aid of an eraser algorithm and the type of interaction of these newly identified lead skeletons by computationally evaluating their interactions with the RALGDS protein^29^. This procedure helps in the discovery of possible lead compounds for pancreatic cancer treatment. Analysing these inhibitors’ pharmacokinetic characteristics is crucial after docking experiments. To determine whether a drug is appropriate for a given therapeutic use, dosage, and possible adverse effects, pharmacokinetics evaluates the drug’s absorption, distribution, metabolism, and excretion in the body^22^. Molecular docking and pharmacokinetic analysis are combined in this integrated strategy to evaluate the safety and efficacy of newly identified lead skeletons as possible therapeutics. The discovery of oncogenes linked to rodent sarcoma virus genes, including Kirsten Rat Sarcoma Virus and Murine Sarcoma Virus, began the quest towards understanding KRAS^30^. These oncogenes are homologous to human KRAS, which has been critical in starting a series of signalling molecules that affect cell proliferation, differentiation, chemotaxis, and apoptosis. We shall explore the complex world of KRAS as we go along, including its protein, gene, carcinogenic function, and the exciting opportunities it offers for cancer treatment^31^. A computational strategy that streamlines the drug discovery process by utilizing in silico methods to predict and refine interactions between drug candidates and biological targets. Methods such as molecular docking & molecular dynamics (MD) simulations enable researchers to examine how small molecules interact with proteins, evaluate the stability of these interactions, and investigate their behavior in a dynamic, physiological context. CADD enhances the identification of potential drug candidates, minimises reliance on extensive laboratory experiments, and supports the development of more targeted and cost-effective therapies.

## Material and methodology

### Genomic analysis

KRAS-associated gene data were collected and screened by Database CBioportal Cancer Genomics (https://www.cbioportal.org/). The CBioportal database was utilized to comprehensively examine patients, analyzing both altered and unaltered genes, including mutation amplifications, deep deletions, multiple alterations, and various types of cancer. Additionally, the study predicted copy number alterations (MSK-IMPACT), splice variants of uncertain significance (VUS), and in-frame mutations^32^. Pathway Analysis of Genes Kras-associated genes were studied on the reactome pathway facilitated the identification of gene pathways, distinguished by its high-performance memory capabilities. To achieve this, the over-representation analysis method is segmented into four distinct steps, each employing specific data structures to enhance performance and minimize memory usage. Pathway analysis methods predominantly serve the analysis of omics data acquired from high-throughput technologies. The discussion focuses on the strengths and weaknesses associated with storing data directly in a relational database for in-database analyses. However, relational databases may encounter challenges in managing large-scale omics data due to limitations in scalability and performance when handling complex data structures and extensive datasets. In contrast, the new pathway analysis approach offers a more optimized solution by leveraging tailored data structures for each analysis step^33^. This enhances processing speed and overall performance, leading to more comprehensive and accurate pathway analysis results.

### Proteomics AI and multi-omics analysis

Multi-omics is the integrated application of various high-throughput screening technologies, including genomics, transcriptomics, single-cell transcriptomics, proteomics, metabolomics, and spatial transcriptomics. These approaches significantly advance disease research^34^.

$${\text{Dintegrated = }}\sum\nolimits_{{i = 1}}^{n} {wi \times Di{\text{ }}}$$

Where D*i*​ represents the dataset from omics source *i*, and *wi*​, is the assigned weight for each data type to optimize predictive accuracy. Grid-based cluster algorithms and AI-based (string) databases centred on protein network interaction These grid techniques partition a structure and convert the data space into a finite number of cells^35^. Clusters are sites that have a higher density of related cells. Because the majority of grid-based clustering approaches rely on cell density computations, these algorithms are density-based it was depicted we should identify clusters of protein-protein interaction by using Grib-based algorithms with identified genes was analysed through the cystoscope software and formed a network and highly identified position^36,37^. The multi-omics analysis was calculated on the Oncogenes of KRAS-associate. to study the Protein-protein interaction and analyse the closeness to predict the therapeutic target. The heat map genes and overlap were identified in the Metascape package and were utilized for gene enrichment analysis, aiming to explore the biological processes and molecular activities associated with unique proteins, tissues, and gene ontology protein domains^38^. Proteins were ranked using various methods such as molecular function, biological process, SMART protein domains, and UniProt annotation by analysing the log ratio of proteins’ Pearson’s r value and p-value^39,40^. Notably, the RALGDS is a potential target for KRAS-associated genes.

### RALGDS lead design and fabrication

In the study of RALGDS interactions, the Schrodinger Maestro software package was used (Version 13.9.132). The protein RALGDS was initially prepared as the source entry, undergoing preprocessing steps that involved adjusting hydrogen atoms and assigning bond orders via the CCD database. A zero-order method was used for the generation of metal-disulfide bonds. With the pH set to 2.0, Epik was utilized to generate the heteroatom states, while the PROPKA algorithm optimized the structure by orienting sample water molecules to assign hydrogen bonds accurately. Subsequently, the protein was minimized using the OPLS4 force field with a heavy atom RMSD convergence set at 0.30 Å. To identify the ligand binding pocket, site map detection was performed using the eraser algorithm. The receptor grid was generated based on the D score and boundary box constructed surrounding the binding site, which identified the location as a potential binding pocket. The scaling VdW radius was set to 1.0, and a cutoff of 0.25 was used for partial charges. The enclosing box was centred on the peptide centroid within the workspace, with the docking peptide length specified at 14 Å^41,42^. With 4 A crop site maps at the nearest site point, the Site Map methodology was cast off to spot the binding pocket in the protein standard grid. To dock the ligand an allosteric grid is built based on the site map locations. Ligand designing and fabrication were conducted by protein-based pharmacophore modelling, utilizing active site ligand properties to detect features such as hydrogen bond donors, acceptors, aromatic and aliphatic. A scaling factor of 0.80 and a partial charge cutoff of 0.15 for van der Waals radii were applied to establish the ligand docking technique. Following the import of the grid file, flexible ligand sampling and extra precision XP setup were executed. This process included sampling nitrogen inversion, considering rigid conformations, and utilizing all predefined functional clusters to bias torsion samples. Epik state penalties remained incorporated into the final dock score. Additionally, the energy window for ring sampling was static at 2.5 kcal/mol for conformer generation, and a minimization distance-dependent dielectric constant value of 2.0 was employed.

### MD simulation studies

Using this technique, the best-identified docked result and MMGBSA result studies are conducted for additional confirmation to investigate the physical conformational changes and set the solvated models. The complex was first solvated using the buffer box size scheming technique and the pre-defined SPC solvent model, with the limit condition set to orthorhombic box shape. Na+ & salt-negative ions (Cl−) were added to the system to neutralise it and reduce the last capacity based on the complex protein’s surface occupation. Molecular dynamic simulation was applied to the solvated type. The trajectory recording interval was set to 100 ps and the simulation time was set to 100 ns with a 1.2 energy gap^43–45^. An entire 1000 frames are produced at a temperature of the NPT ensemble class of 300 k with 1.01325 bar pressure, with the model relaxed earlier to simulation The last comprehensive simulation, with trajectory quality ensured, underwent analysis with a block length averaging 10.0 ps^46,47^. This protocol for quality analysis precise the 100 ns simulation, presenting graphs of Total Energy (TE) (kcal/mol), Potential Energy (PE) (kcal/mol), Temperature (T) (K), Pressure (P) (bar), and Volume (P) (Å^3^) parameters. The Trajectory was analysed by using the GPA accelerated Desmond package. Throughout the 100ns period times, they observed the RMSD, and RMSF by using a mathematical model and also analysed the protein-ligand contacts, ligand-protein contacts, and heat map interaction^48^.

1 $${\text{RMSD}} = \sum\nolimits_{{i = 1}}^{N} {(xi - x1)/N}$$

2 $${\text{RMSF}} = \sqrt {1/N\sum\limits_{j}^{N} {\left( {xi\left( j \right) - \left( {xi} \right)} \right)2} }$$

### ADMET prediction

ADMET (Absorption, Distribution, Metabolism, Excretion, and Toxicity) forecasts were shown using the Graph-based signature approach. Understanding a compound’s pharmacokinetics is crucial for defining its drug-likeness. Values from medicines with comparable in vivo experimental data were utilized to assess query structures intended for drug discovery^49^. The tool receives the canonical form of the lead compound as input and incorporates various parameters such as lipophilicity, polarity, molecular weight, saturation, and insolubility. Furthermore, the tool provides bioavailability scores, drug-likeness assessments, adherence to the Lipinski rule of 5, & toxicity evaluations, amongst other parameters^50,51^. These data collectively contribute to the assessment of lead compound properties, aiding in the identification of promising candidates for drug development.

## Result and discussion

### Genomic analysis

**Fig. 1:**
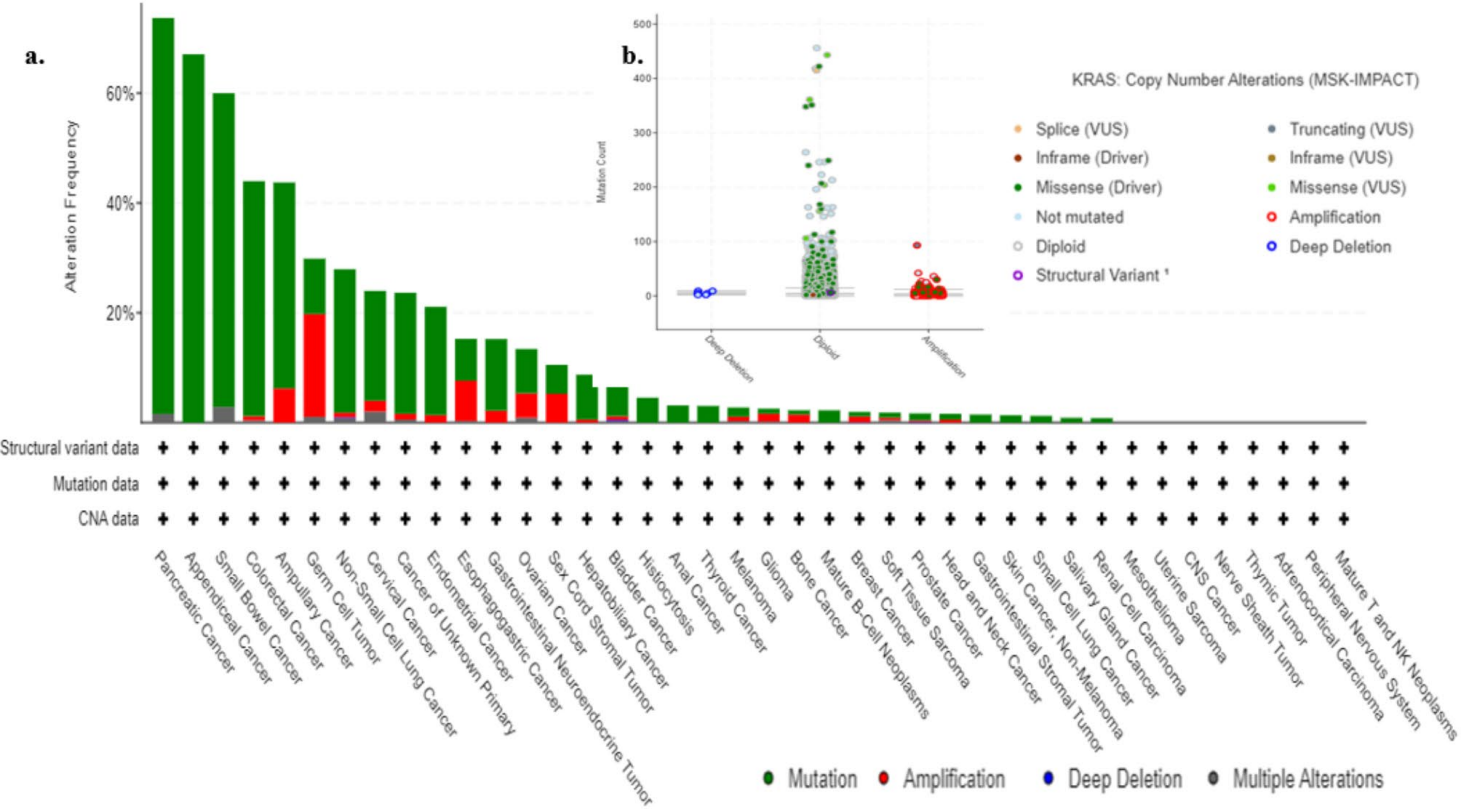
(**a**) KRAS Frequency alteration and types of cancer analysis (**b**) Copy number alteration of KRAS gene.

Genomic analysis conducted using CBioPortal for cancer genomics focused on the KRAS gene. The most common alteration observed was KRAS mutations, followed by amplifications. These alterations were predominantly found in pancreatic cancer. The KRAS gene is implicated in approximately 40 different types of cancers. It is illustrated in Fig. 1A. The genomic analysis of KRAS copy number alterations using CBioPortal sheds light on the significance of these variations in cancer progression. The results show that diploid KRAS genes with missense driver mutations are the most common, with a high log ratio of 480.

This indicates a strong association between diploid KRAS alterations and cancer development, positioning them as potential targets for cancer therapies. Amplifications, though observed less frequently (log ratio of 100), also contain missense driver mutations. While these alterations are not as prevalent as diploid forms, they may still contribute to cancer progression, suggesting that both diploid and amplified KRAS alterations play important roles in driving oncogenesis, albeit through distinct mechanisms^52^. The presence of deep deletions in KRAS, although less common, suggests the involvement of additional, more complex factors in cancer progression. Although deletions typically lead to loss of function, in the case of KRAS, their impact may be less significant compared to diploid or amplified states.

**Fig. 2:**
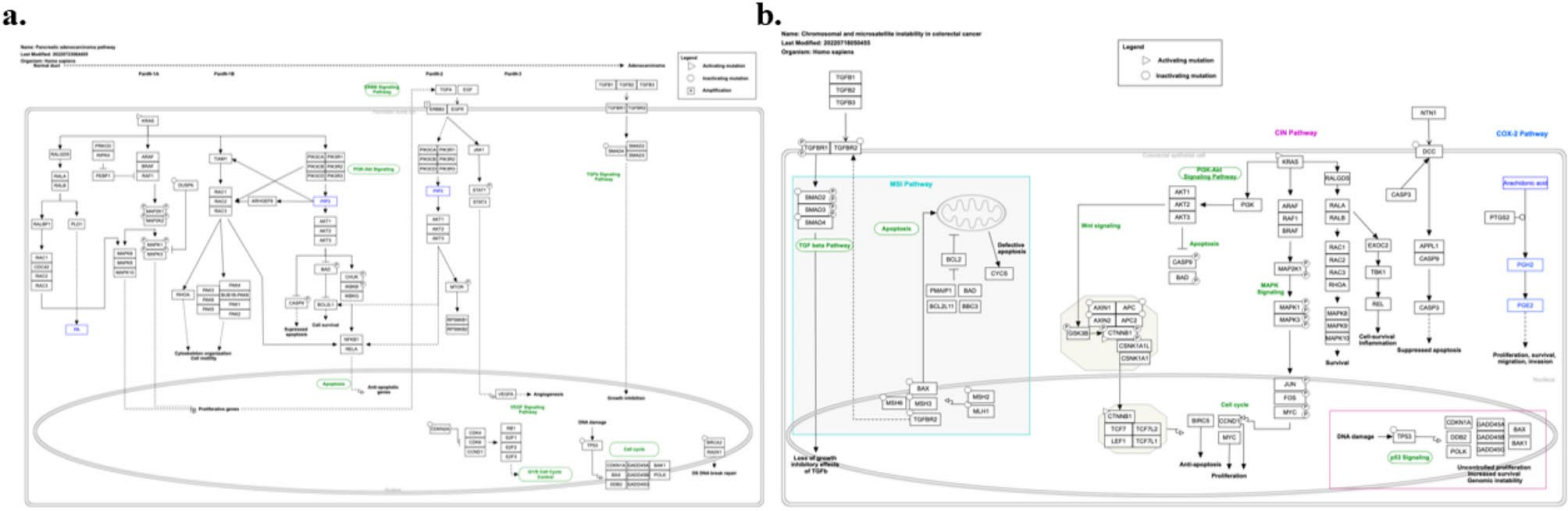
(**a**) Pathway of pancreatic cancer (**b**) Pathway of colorectal cancer. Figure is reproduced from Ref^58,59^.

The pathways were presented majorly with Infiltrating ductal adenocarcinoma is the most predominant form of pancreatic malignancy, often referred to as pancreatic ductal adenocarcinoma (PDA) by researchers^53^. The development of PDA involves a stepwise development from normal ductal epithelium to aggressive cancer, with distinct precursor lesions known as Pancreatic Intraepithelial Neoplasia (PanINs) playing a crucial role in this process^54^. The genetic alterations associated with PDA occur at different stages of this progression. Early in the process, we observe the overexpression of the HER-2/neu (ERBB2) gene and then the occurrence of triggering point mutations in the K-RAS gene. At an intermediate stage, there is inactivation of the p16 gene (CDKN2A). As the disease advances, the inactivation of essential genes like p53, SMAD4, and BRCA2 takes place relatively late in the series of events. These genetic mutations have a deep impact on the development and progression of PDA as illustrated in Fig. 2a. K-RAS, a key player in this situation, triggers various downstream signalling pathways. While it’s common to interpret EGF receptors as upstream activators of RAS proteins, it’s essential to note that they can also function as RAS signal transducers through a mechanism where RAS induces the autocrine initiation of the EGFR group ligands. Furthermore, PDA exhibits wide genomic unpredictability and aneuploidy, which means that the cancer cells have an abnormal number of chromosomes. This genomic instability can be recognized by factors like telomere attrition in addition to mutations in genes like p53 and BRCA2, which probably contribute to these characteristics Additionally, the deactivation of the SMAD4 tumour suppressor gene results in the forfeiture of the inhibitory effect of the transforming growth factor-beta signalling pathway, which is another serious aspect of PDA development. To summarize, the progression of pancreatic ductal adenocarcinoma and the associated genetic mutations follow a defined sequence, as illustrated in Fig. 1b, from left to right^55^. Understanding these molecular events is important for improving our knowledge of PDA and developing more effective diagnostic and treatment strategies CRC initiates since the epithelial cells lining the colon and rectum, and its development involves the buildup of specific genetic modifications in oncogenes in addition to tumour suppressor genes (TSGs). There are two key mechanisms of genomic variability related to the progression of sporadic CRC. The initial is chromosomal instability (CIN), a result of genetic variations that activate oncogenes like K-ras and deactivate TSGs like p53, DCC/Smad4, also APC. Another mechanism is microsatellite instability (MSI), which arises after the deactivation of DNA mismatch repair genes MLH1 or MSH2 due to hypermethylation of their promoters and minor mutations in genes by coding microsatellites, such by way of transforming growth factor receptor II (TGF-RII) in addition BAX^56,57^.

In addition to sporadic cases, there are hereditary syndromes with germline changes in definite genes, such as mutations in the tumour suppressor gene APC on chromosome 5q in ancestral adenomatous polyposis (FAP) and mutated DNA mismatch repair genes in hereditary nonpolyposis colorectal cancer (HNPCC)^60^. The greatest shared mutation in colon cancer includes the deactivation of APC. In cases where APC doesn’t have a deactivating mutation, there are frequently triggering mutations in β-catenin. To develop cancer, together alleles must change. When carriers have APC deactivating mutations, the danger of colorectal tumours at age 40 is nearly 100%^61^. The influence of KRAS alterations deeply depends on the order in which mutations occur. Main KRAS changes usually result in self-limiting hyperplastic or marginal lesions, nevertheless, if they happen afterward a previous APC mutation, they often evolve into cancer. KRAS transformation is also a strong forecaster of deprived response to panitumumab and cetuximab treatment in CRC^62^. It is depicted in Fig. 2b. Testing for “activating” mutations in the KRAS gene is currently the most reliable way to forecast a colorectal cancer patient’s response to EGFR-inhibiting medications. Loss of heterozygosity (LOH) of DCC in area 18q21 is one of the greatest recurrent genetic irregularities in advanced CRC furthermore, TGFβ signalling is active in nearly CRC cells with MSI alterations in TGFBR2, occurring in 30–50% of CRC. Individuals whose tumours direct the altered version of the KRAS gene do not answer to cetuximab or panitumumab. DCC is a gene with a conditional role, acting as both a tumour suppressor and an oncogene. Once DCC exists and is not triggered by netrin, it promotes programmed cell death and represses tumour formation^63^. However, once DCC exists and netrin-activated, it supports cell survival and stands in as an oncoprotein. Abnormal overexpression of cyclooxygenase-2 (COX-2) is also understood to play a significant part in CRC growth. The tumorigenic properties of COX-2 are recognized in the generation of PGE2 (prostaglandin E2), and enhanced ranges of PGE2 have been observed in colorectal adenomas and carcinomas. COX-2 in addition to PGE2 regulates various processes in colorectal tumours, including proliferation, survival, migration, and invasion.” This elaboration provides a more comprehensive understanding of the genetic and molecular factors contributing to colorectal cancer development and progression.

### Proteomics AI and multi-omics analysis

Incorporating AI into network pharmacology provides two primary advantages through a deep interpretable network relationship inference framework. First, the hierarchical structure and biologically relevant nodes allow for interpretable feature learning, giving biological context to the model’s predictions. Second, this approach expands the feature space by learning both the network’s features and their associations, effectively addressing the challenges of low generalization and limited interpretability commonly faced by AI-based models in biological networks. Additionally, advancements in language model technologies, particularly large language models (LLMs), have helped mitigate previous constraints related to computational resources and data volume. These developments facilitate the conception of robust deep interpretable models, streamlining network pharmacology methodologies and enhancing the utility of AI in this domain.

**Fig. 3:**
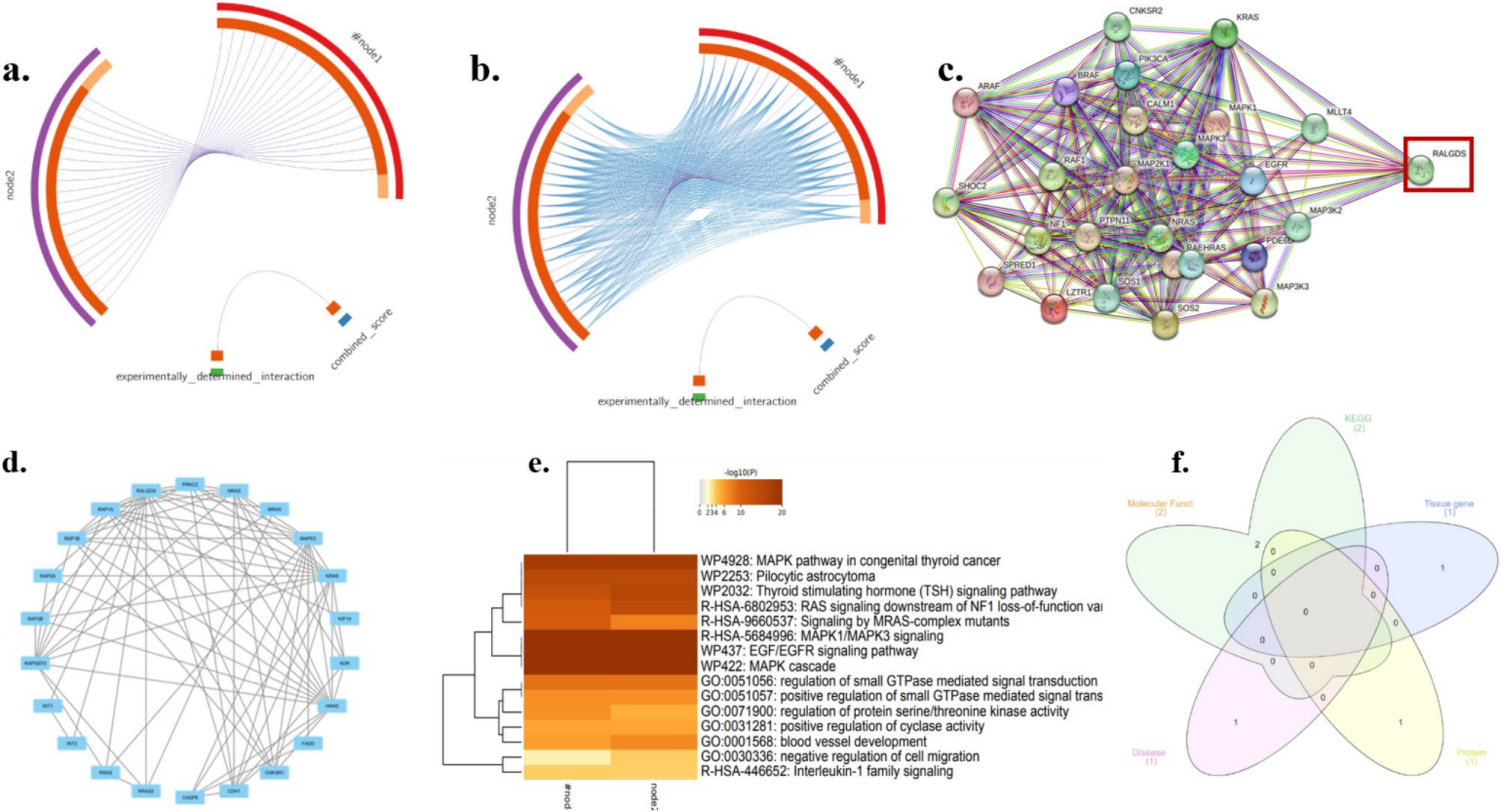
(**a**) and (**b**) KRAS associated overlapped genes similarity (**c**) and (**d**) Network pharmacology of KRAS-associated genes (**e**) Heat map of gene enrichment KRAS (**f**) components of gene enrichment.

Figure 3a illustrates the gene level, identical genes are linked by purple curves. Figure 3b at the common term level, blue curves connect genes fitting to the similar enriched ontology term, revealing functional similarities. The internal circle signifies gene lists, with dark orange indicating genes present in numerous lists in addition to light orange denoting genes exclusive to a specific list. This visual analysis provides insights into both genetic overlap and shared biological functions, providing an understanding of the relationships between the gene sets^64^. The AI proteomics analysis was done on the Strings db database to identify the protein-protein interaction and identify the nodes as illustrated in Fig. 3c. As a result, the KRAS genes have high nodes and closeness was found in the RALGDS gene marked in Fig. 3d. Moreover, we examined and projected gene overlaps, heat maps, and protein enrichment analyses such as molecular function, tissue gene, protein domain, KEGG pathway, and disease association to further validate^65^. The protein network cluster has 23 proteins - protein has interacted strongly and a large number of nodes in the RALGDS protein, which was predicted on the cystoscope. It illustrates the comparison of two gene lists, showing overlap at two levels^66^. The heatmap of gene enrichment analysis was done on the Metascape software to predict the pathway of the genes based on the log ratio. From 0 to 20, they are mostly the highest signalling RAL guanine nucleotide dissociation stimulator (RALGDS) functions as a key downstream effector in KRAS signaling, acting as a GTP/GDP exchange factor to promote the GDP-GTP conversion for RAS-like (RAL) proteins. This activation of RAL GTPases by RALGDS contributes significantly to pro-survival mechanisms, supporting cellular proliferation and cell cycle progression. Recent studies highlight RALGDS’s essential role in modulating KRAS-driven oncogenic pathways, linking RALGDS activity to enhanced tumour growth and metastasis in various cancers. RALGDS thus emerges as a critical mediator in KRAS signalling, with potential therapeutic targeting implications^21,67^. Ongoing research is centered on exploring the mechanistic pathways and regulatory interactions of RALGDS within the KRAS network, aiming to identify novel intervention strategies targeting the MAPK cascade and EGFR signaling pathways. Understanding these interactions is crucial, as they play a significant role in cancer progression and therapeutic resistance. In addition, a predictive analysis was conducted to identify genes associated with thyroid cancer, with the results visualized in Fig. 3e.

**Fig. 4:**
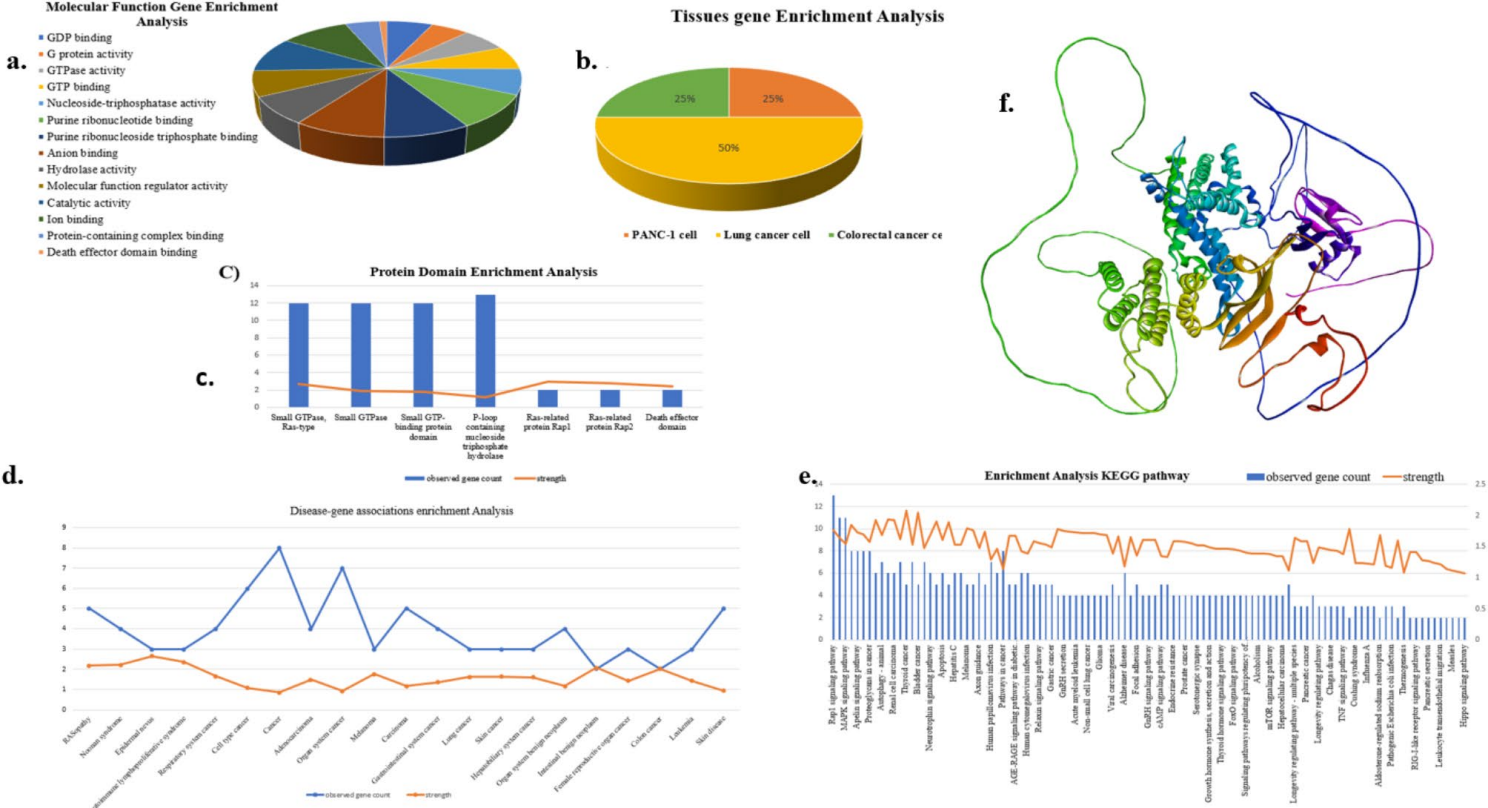
Enrichment analysis (**a**) Molecular function (**b**) tissue gene (**c**) protein domain (**d**) Disease associate enrichment analysis (**e**) KEEG enrichment analysis (**f**) RALDGS protein.

The omics analysis was performed on the major five components of the matching proteins which are represented in Fig. 4 as a molecular function, tissue gene, protein domain, and disease-associated KEGG pathway^68^. We also identified the matching proteins KRAS set of RALGDS protein in the analysis and also identified the genes associated with function strength and count. Disease, molecular function, proteins, KEGG, and tissues are the five primary components of enrichment analysis. These are represented in Fig. 4. The Molecular Function component shows 14 available components, illustrated in Fig. 4a. The major components include Anion binding, with matching proteins found to be RAP2A, RRAS, RAP1B, KRAS, RRAS2, MAPK3, KDR, RAPGEF2, MRAS, RAP2B, RIT2, KIF14, RIT1, NRAS, RALGDS, RAP1A, PRKCZ, and HRAS. The purine ribonucleoside triphosphate binding component matches proteins such as RAP2A, RRAS, RAP1B, KRAS, RRAS2, MAPK3, KDR, MRAS, RAP2B, RIT2, KIF14, RIT1, NRAS, RAP1A, PRKCZ, and HRAS. Similarly, the purine nucleotide binding component corresponds to proteins like RAP2A, RRAS, RAP1B, KRAS, RRAS2, MAPK3, KDR, RAPGEF2, MRAS, RAP2B, RIT2, KIF14, RIT1, NRAS, RAP1A, PRKCZ, and HRAS. These three components are majorly present in the molecular function enrichment analysis. The tissue enrichment analysis unveiled three distinct cancer types, notably showcasing lung cancer’s dominance, encompassing about 50% of the tissues examined. Among the discerned proteins, KRAS, CDH1, NRAS, and HRAS emerged as key players within this context. Additionally, pancreatic and colorectal cancers emerged within the same tissue milieu, featuring the prominent presence of KRAS and CDH1 proteins. This insightful data is eloquently depicted in Fig. 4b. and In Fig. 4c, the protein domain enrichment analysis reveals a notable emphasis on the P-loop containing nucleoside triphosphate hydrolase. This domain garnered particular prominence, showcasing a rich abundance of proteins including RAP2A, RRAS, RAP1B, KRAS, RRAS2, MRAS, RAP2B, RIT2, KIF14, RIT1, NRAS, RAP1A, RALGDS and HRAS. This insightful data underscores the intricate molecular landscape under scrutiny, examined proteins. The analysis of the KEGG pathway revealed a comprehensive array of 104 components, prominently featuring pathways such as Rap1 signalling, MAPK1 signalling, and several cancer signalling pathways. Within these pathways, a cohort of matching proteins was identified, including RRAS, RAP1B, KRAS, CDH1, MAPK3, KDR, RAPGEF2, MRAS, NRAS, RAP1A, RALGDS, PRKCZ, and HRAS. This extensive repertoire of proteins underscores their crucial roles in modulating various cellular processes and highlights their significance in the context of KEGG pathway analysis. The disease enrichment analysis revealed a significant gene count associated with cancer, indicating a substantial presence of genes implicated in various types of cancer. This comprehensive analysis, depicted in Fig. 4d, illuminated the diverse spectrum of cancer types involved. Notably, matching proteins such as KRAS, RRAS2, CDH1, KDR, CASP8, RALGDS, RIT1, NRAS, and HRAS were identified within this enriched Analysis as shown in Fig. 4e. proteins KRAS, NRAS, and HRAS are crucial components of the RAS signaling pathway, which regulates cell proliferation, differentiation, and survival. Mutations in these proteins often result in their continuous activation, leading to uncontrolled cell growth and cancer progression. The role of CDH1, which is involved in cell-cell adhesion, is particularly important in preventing cancer metastasis; loss of its function can contribute to tumor invasion and metastasis. CASP8, on the other hand, plays a key role in apoptosis, and its dysregulation can lead to evasion of programmed cell death, thus promoting cancer cell survival. KDR (VEGFR-2) is a vital mediator in angiogenesis, the process by which new blood vessels form to supply nutrients to growing tumors. By targeting KDR, it may be possible to inhibit tumor vascularization and slow cancer growth. Additionally, proteins such as RALGDS and RRAS2 are involved in regulating cell signaling related to proliferation and differentiation, which can influence tumor development and progression The abundance of these proteins underscores their potential roles in the pathogenesis and progression of cancer. RALDGS protein is proposed as a versatile guanine nucleotide exchange factor (GEF), triggering RalA and RalB GTPases to orchestrate intracellular transport^69^. Acting as an effector for R-Ras, H-Ras, K-Ras, and Rap, RALDGS plays a significant role in transducing signals downstream of these GTPases, influencing cellular processes and In vivo studies ana Gonzalez et al. (2005) experimentally proved the formation of cancer in mouse model in Fig. 4f. This multifaceted protein’s interactions and regulatory functions contribute to the coordination of intracellular transport and the maintenance of cellular homeostasis, describing its significance in fundamental cellular processes.

### RALGDS lead design and fabrication

**Fig. 5:**
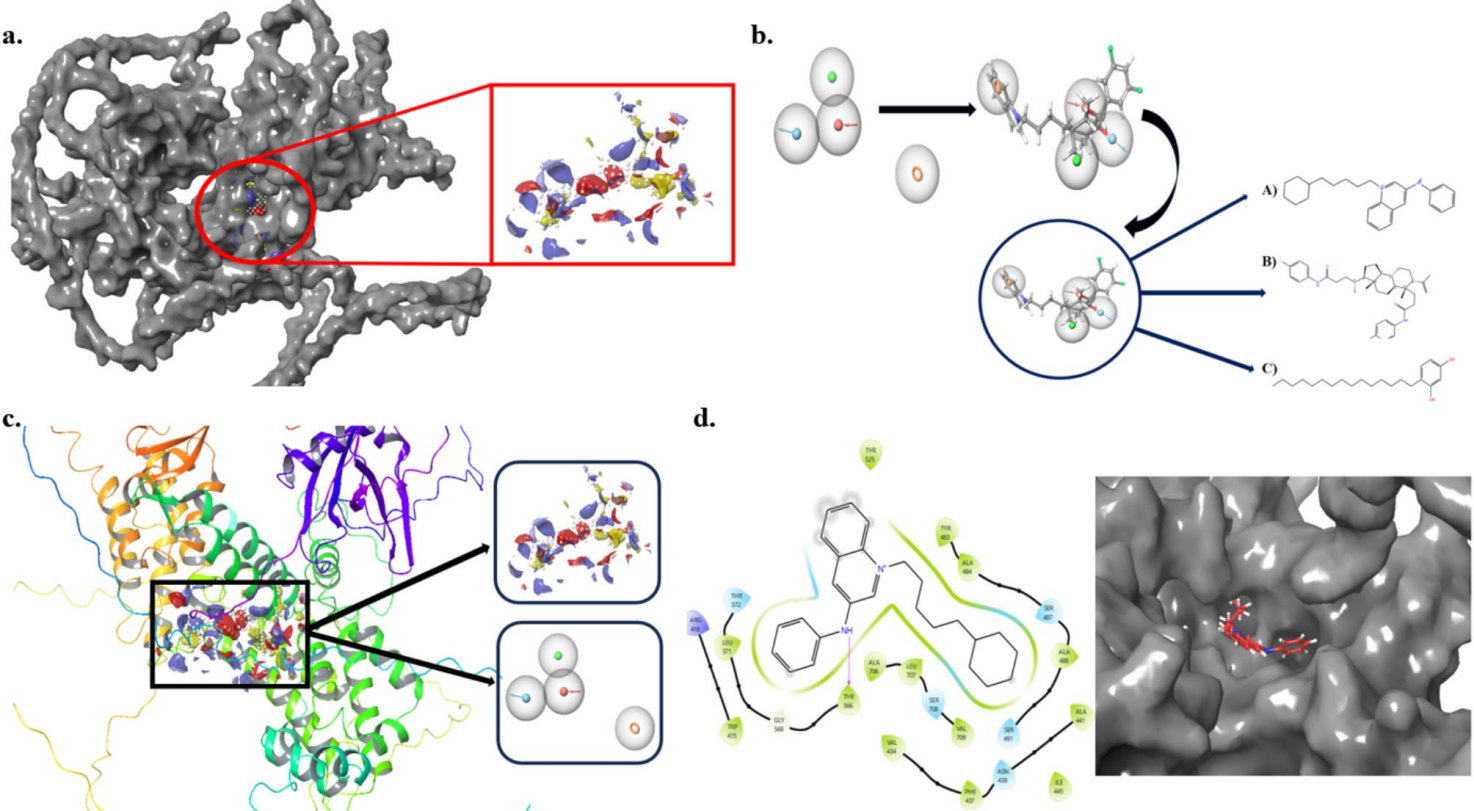
(**a**) Binding pocket of RALDGS (**b**) RALDGS based on pharmacophore to lead generation (**c**) Binding pocket of RALDGS based on pharmacophore (**d**) Lead Interaction.

The Alpha fold protein AF-Q12967 does not contain a catalytic pocket ligand, hence, we performed the binding pocket analysis in the maestro Schrodinger package using the binding pocket prediction module. First, the protein was prepared, pre-processed, removed water, and minimized and given the command binding site detection to identify the binding site it was illustrated in Fig. 5a^68,70^. Binding site selection was identified by the D score which was greater than 1, so it confirmed the organic compound binding protein. In Fig. 5c Ligand ASL properties were predicted for the pharmacophore developing (chain. name A (res.num 398 | res.num 401 | res.num 415 | res.num 416 | res.num 418 | res.num 419 | res.num 420 | res.num 433 | res.num 434 | res.num 436 | res.num 437 | res.num 438 | res.num 480 | res.num 483 | res.num 519 | res.num 524 | res.num 525 | res.num 529 | res.num 565 | res.num 566 | res.num 567 | res.num 568 | res.num 569 | res.num 571 | res.num 572 | res.num 575 | res.num 709 | res.num 711 | res.num 712 | res.num 713 | res.num 714)) by the that the forming of the pharmacophore developed the molecule with proof H-Bond donor & acceptor and aromatic system.

The nature of binding pocket analysis showed the Hydrophobic residues (Val, Leu, Phe, Met) stabilize the protein structure and fold to form binding pockets, and the Polar, charged residues (Ser, Thr, His, Asp, Arg, Lys) aid to substrate binding, and act as a potentially catalytic amino acid. The aromatic residues (Tyr, Trp) contribute to structural stability and ligand recognition. Two additional flexible residues (Gly, Pro) facilitate structural adjustments to form proper configurational ligand binding.

We designed an extensive set of molecules and analyzed their pharmacophoric features to identify three lead compounds, as illustrated in Fig. 5b. These molecules were developed based on the E-pharmacophore model, which included four key features: aromatic rings, hydrogen bond donors, and acceptors. During molecular docking, it was observed that the octyl aromatic ring system of one of the selected molecules fit well within the protein’s binding cavity. The docking score, along with the MMGBSA binding energy calculation, yielded a value of -53.53 kcal/mol. The 2D interaction map is depicted in Fig. 5d, with additional details in Supplementary Fig. 1. The binding interactions revealed one hydrogen bond with residue TYR 566. Hydrophobic interactions were noted with several amino acids, including TYR 524, TYR 483, ALA 484, ALA 488, ALA 441, ILE 445, PHE 437, VAL 434, VAL 709, ALA 706, LEU 707, and TRP 415. Additionally, polar interactions were observed with residues ASN 438, SER 491, SER 708, SER 487, and THR 572. A positively charged interaction was identified with ARG 418. These results, including the MMGBSA it was depicted in (Table 1) The polar solvation energy, calculated using the Generalized Born model, was balanced effectively by the non-polar solvation contributions, which were optimized by the hydrophobic interactions observed with residues are ALA 484, TYR 483, and PHE 437.

**Table 1:** Docking score & MMGBSA score of the lead molecule.

Lead molecule	Glide score (Kcal/mol)	MMGBSA (Kcal/mol)
A	− 6.026	− 53.53
B	− 5.928	− 51.06
C	− 5.260	− 36.66

### MD simulation studies

**Fig. 6:**
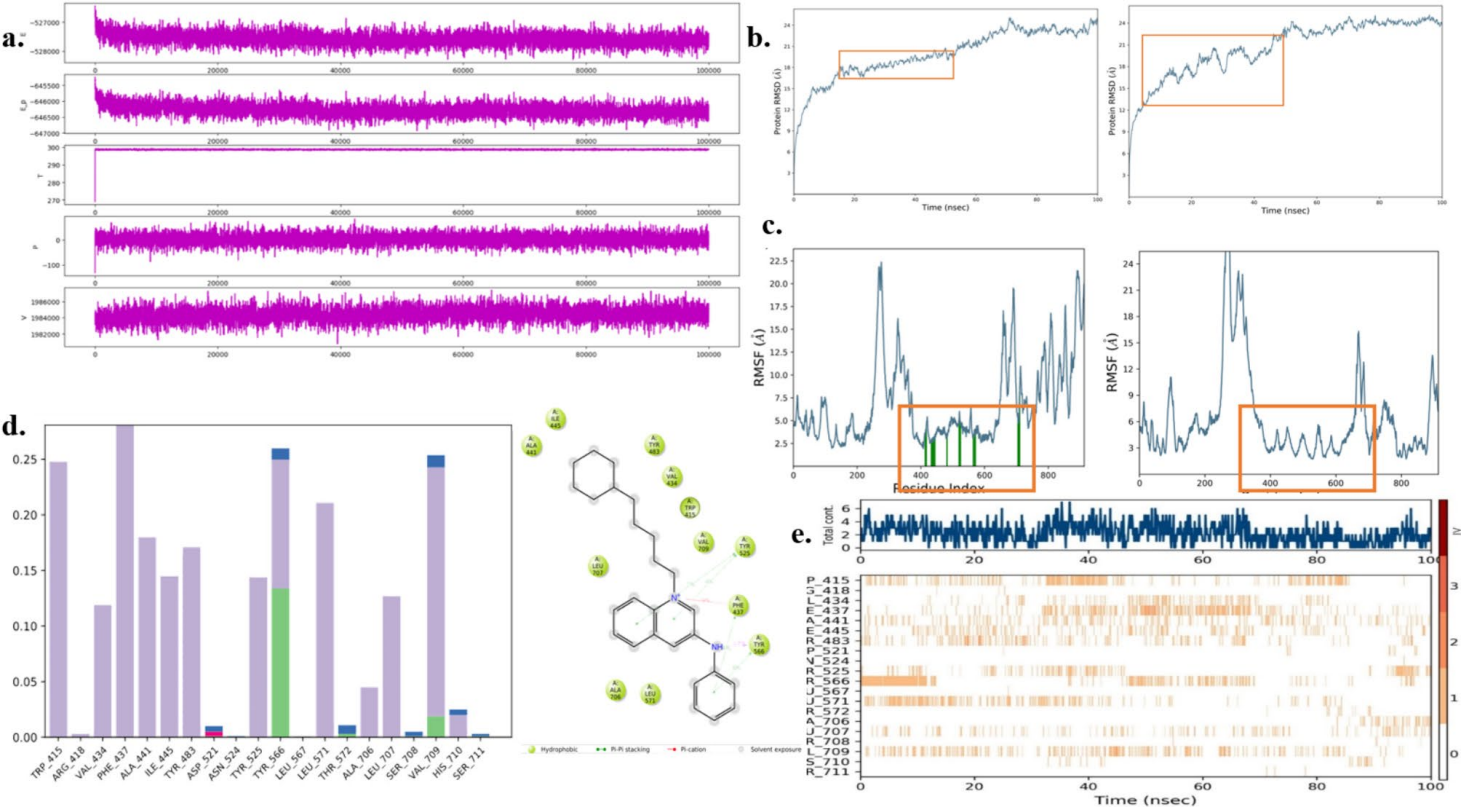
(**a**) Quality Simulation Analysis 100 ns (**b**) RMSD fluctuation lead and protein (**c**) RMS fluctuation protein-ligand complex and protein (**d**) Interaction of protein-ligand contacts 100 ns time and Interaction map of interaction (**e**) Heat map 100ns protein complex interaction.

The quality of the molecular dynamic simulation results was thoroughly assessed, and the limit values found were illustrated in Fig. 6a; Table 2 to be within desirable ranges, indicating a high level of reliability and accuracy. Specifically, the multifaceted simulation exhibited stable volume (V), pressure (P), temperature (T), potential energy (PE), and overall energy profiles throughout the simulation.

**Table 2:** Parameters of MD simulation quality analysis.

Properties	Average	Std. Dev	Slope (ps^− 1^)
Quality values of NRPB1 protein complex simulation (100ns)			
Overall energy (kcal/mol)	− 527580.359	210.757	− 0.002
PE (kcal/mol)	− 646278.828	184.545	− 0.002
T (K)	298.707	0.378	0.000
P (bar)	1.183	21.049	0.000
V (A3)	1984368.644	863.914	0.005

The RMSD graph was obtained from the 100ns molecular dynamics (MD) simulation, as illustrated in Fig. 6b. showcased the behavior of the protein-ligand complex. It was observed that the protein-ligand complex exhibited fluctuations within the range of 1–2 angstroms, indicating relatively stable behavior compared to the protein alone. Conversely, the protein alone demonstrated greater fluctuations, surpassing 3 angstroms, suggesting less stability in isolation. In conclusion, the analysis reveals that the protein-ligand complex, particularly involving the RALGDS protein, maintains stability throughout the MD simulation.

The protein Root Mean Square Fluctuation (RMSF) analysis revealed that the protein-ligand complex exhibited reduced fluctuation, measuring approximately 22.5 angstroms, compared to the plain protein which displayed higher fluctuation at around 25 angstroms. Moreover, the interaction analysis revealed that amino acid interactions between the ligand molecule and the protein occurred consistently from frames 400 to 700 of the simulation. This time frame indicates the formation and stability of interactions between the ligand and the protein residues. These findings are visually represented in Fig. 6c. These interactions primarily involved π–π stacked interactions and H-bond interactions with active amino acids, notably TYR 525 and PHE 437 as illustrated in Fig. 6d. The heat map analysis performed throughout the simulation period revealed significant interactions between the ligand and the protein, with particularly strong involvement of residue TYR 566, as depicted in the accompanying. The analysis indicated that more than 20 amino acid interactions were maintained consistently throughout the simulation, as shown in Fig. 6e. These interactions highlight the stability and robustness of the binding. By the end of the simulation, it was evident that the newly designed octyl aromatic ring system demonstrated stable binding within the pocket of the RALGDS protein.

### ADMET prediction

The ADMET profile was determined using the PKCSM database, allowing us to pinpoint three leads for comparison. We verified parameters such as water solubility, Caco2 permeability, intestinal absorption, and skin permeability. Through this analysis, lead 2 emerged as having excellent absorption capabilities^71^. When it comes to P-glycoprotein substrate, these leads demonstrated no inhibitory effects. Specifically, for P-glycoprotein I and II inhibitors, lead 3 displayed no inhibitory effect, lead 1 is a substrate of CYP2D6 and CYP3A4 enzymes and functions as an inhibitor of CYP1A2, CYP2D6, and CYP3A4. Lead 2, on the other hand, is only a substrate for CYP3A4 and does not inhibit any of the listed cytochrome p450 enzymes.

**Fig. 7:**
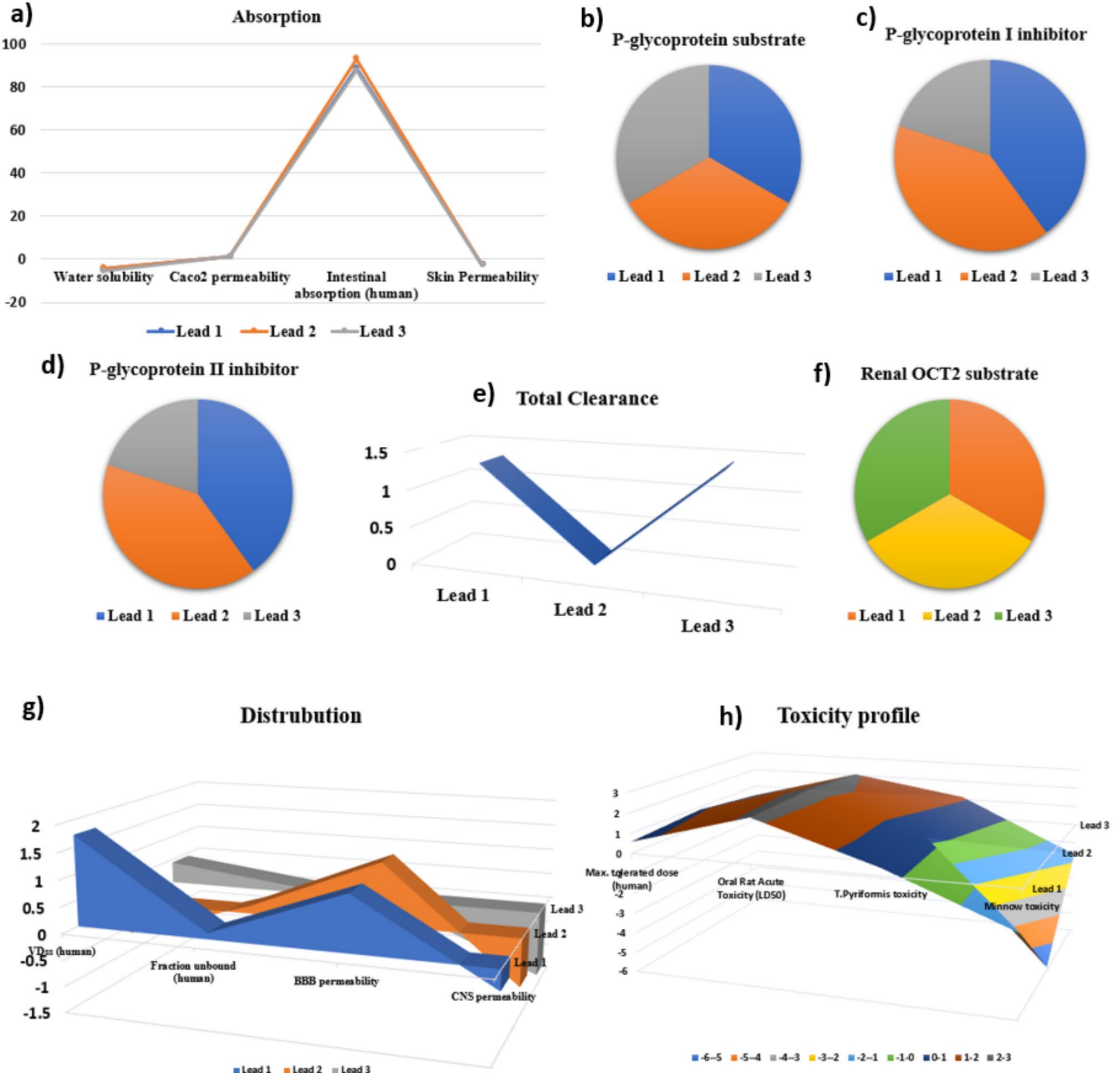
(**a**) Absorption (**b**) Glycoprotein substrate (**c**) Glycoprotein I Inhibitors (**d**) Glycoprotein II Inhibitors (**e**) Total clearance (**f**) RENAL OCT2 (**g**) Distribution (**h**) Lead molecule toxicity profile.

Lead 3 acts as a substrate for CYP3A4 and inhibits CYP1A2 but does not inhibit CYP2D6 Comparing these properties, lead 1 stands out for its dual substrate activity with CYP2D6 and CYP3A4 and broad inhibition profile, while lead 2 primarily serves as a substrate for CYP3A4 without inhibitory effects In Fig. 7c. Lead 3 shares substrate activity with cyp3a4 but differs in its selective inhibition of CYP1A2 only in supplementary 1. The ADMET (absorption, distribution, metabolism, excretion, and toxicity) properties of three lead compounds (lead 1, lead 2, and lead 3) reveal distinct profiles. All leads exhibit similar absorption characteristics in terms of water solubility, caco-2 permeability, intestinal absorption in humans, and skin permeability. Lead 3 is the most likely to be a substrate for p-glycoprotein and also the most potent inhibitor of p-glycoprotein (i) while lead 1 shows the highest inhibitory activity for p-glycoprotein (ii) Regarding total clearance, lead 1 is eliminated the fastest, followed by lead 3 and lead 2. In terms of renal oct2 substrate likelihood, lead 3 is the most probable, followed by lead 2 and lead 1. This comprehensive analysis indicates varying degrees of absorption and interaction as shown in Figure A with p-glycoprotein, clearance rates, and renal transport among the three leads, providing valuable insights for further development. The provided image features two 3D graphs comparing three leads (Lead 1, Lead 2, and Lead 3) on distribution and toxicity profiles as depicted in Figures d, e &f. Graph A assesses the volume of distribution (Vdss), fraction unbound in humans, blood-brain barrier (BBB) permeability, and central nervous system (CNS) permeability. Graph B evaluates the maximum tolerated dose in humans, oral rat acute toxicity (LD50), and toxicity in Tetrahymena pyriformis and minnows. Lead 1 shows good CNS penetration but high environmental toxicity. In Figure h Lead 2 presents a balanced profile with moderate distribution and better safety. Lead 3 exhibits strong BBB and CNS permeability but poor overall distribution and lower safety. Based on these attributes.

## Conclusion

This research study employed an Artificial Intelligence-driven network biology approach to investigate the involvement of KRAS Oncogenes in diverse cancer types. By joining genomics statistics and accessing comprehensive genetic datasets from CBioportal cancer genomics, we developed an innovative drug discovery methodology. Through meticulous gene analysis encompassing Pathways, omics, and enrichment analyses, to study the biological function and molecular function of the protein we found that the RALGDS protein is a promising biomarker linked to KRAS. Following the modeling of the RALGDS protein, we utilized the eraser algorithm to identify the optimal binding cavity. Subsequently, we developed the single protein E-pharmacophore properties to design a molecule based on the ASL properties of the binding cavity. Upon docking the designed molecule, we obtained an MMGBSA score of -53.53 Kcal/mol and the octyl aromatic ring lead 1 has the best ADMET profile providing further validation. To enhance confirmation, we conducted Molecular Dynamics simulations to analyse the stability of the protein lead complex. Our findings indicate the formation of a stable complex, affirming the efficacy of the proposed molecule in RALGDS.

## Electronic supplementary material

Below is the link to the electronic supplementary material.

Supplementary Material 1

Supplementary Material 2

## Data Availability

Data is provided within the manuscript or supplementary information files.
