# Supplementary material for: Network pharmacology and AI in cancer research uncovering biomarkers and therapeutic targets for RALGDS mutations
Source: Sci Rep. 2025 Mar 29;15:10938. doi: 10.1038/s41598-025-91568-x (PMC11954960; doi:10.1038/s41598-025-91568-x)
Supplement: Supplementary file 1 — Supplementary Material 1 [file 41598_2025_91568_MOESM1_ESM.docx]

**Network Pharmacology and AI in Cancer Research Uncovering Biomarkers and Therapeutic Targets for RALGDS Mutations**

**S. Mohammed Zaidh^1,2^, Hariharan Thirumalai Vengateswaran^1^, Kiran Balasaheb Aher^3^, Girija Balasaheb Bhavar^4^, Irfan N^1*^, Mohammad Habeeb^1^,** **K.N.V. Chenchu Lakshmi^5^**

**Supplementary**


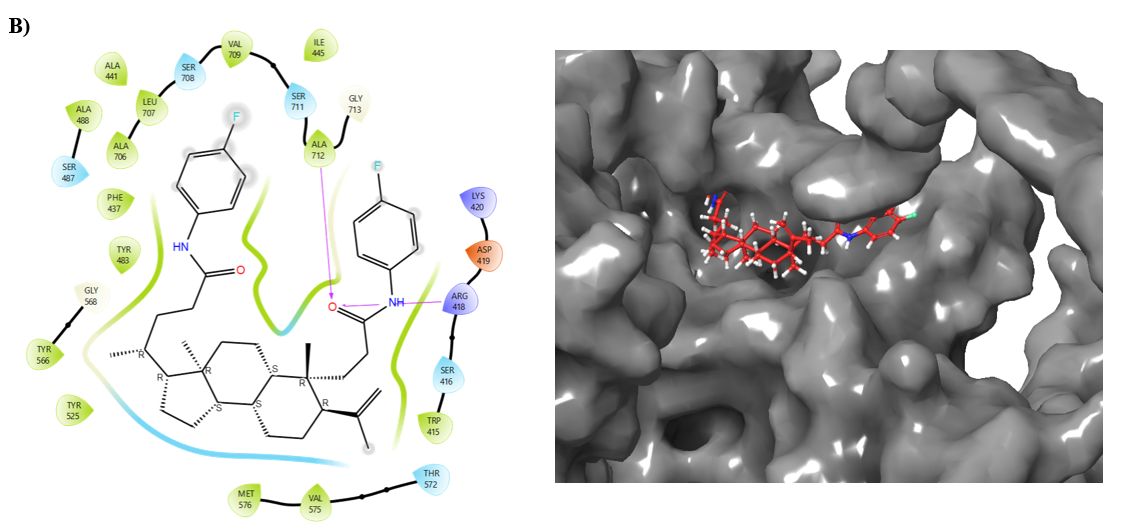


*Fig 1* Additional Ligand interacted with RALGDS

The Fig 1 B & S2 illustrates the 1 H-bond interaction with Active amino acids of ALA 712, ARG 418. And MMGBSA score was found -51.06. The hydrophobic has ILE 445, VAL 709, ALA 441, LEU 707, ALA 488, ALA 706, PHE 437, TYR 483, TYR 566, TYR 525, MET 576, VAL 575, TRP 415. The polar has SER 711, SER 708, SER 487, SER 416, THR 572. The positive charge has ARG 418, LYS 420 and negative charge has ASP 419.


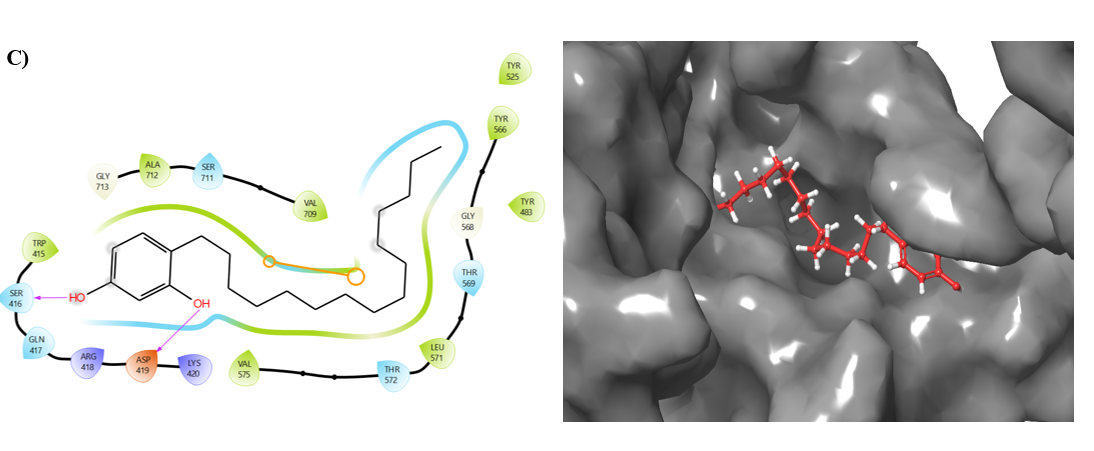


**Fig 2** Additional2 Ligand for RALGDS interaction

In Fig 2 & S3 has H-bond interaction SER 416, ASP 419 and found the MMGBSA -36.66 the hydrophobic has TYR 525, TYR 566, TYR 483, LEU 571, LEU 575, TRP 415, ALA 712, VAL 709. The polar has SER 711, GLN 417, THR 572, THR 569. The positive charge has ARG 418, LYS 420. The negative charge has ASP 419.

**ADMET**


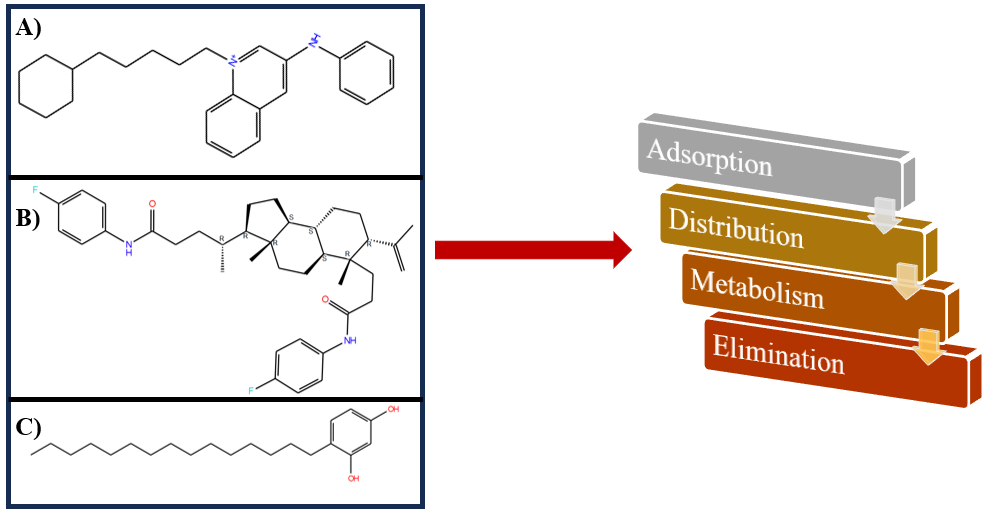


**Table** 3 Metabolism Lead molecules

| **Properties name** | **Lead 1** | **Lead 2** | **Lead 3** |
| --- | --- | --- | --- |
| CYP2D6 substrate | Yes | No | No |
| CYP3A4 substrate | Yes | Yes | Yes |
| CYP1A2 inhibitor | Yes | No | Yes |
| CYP2C19 inhibitor | No | No | No |
| CYP2C9 inhibitor | No | No | No |
| CYP2D6 inhibitor | Yes | No | No |
| CYP3A4 inhibitor | Yes | No | No |
