# Supplementary material for: Network pharmacology and AI in cancer research uncovering biomarkers and therapeutic targets for RALGDS mutations
Source: Sci Rep. 2025 Mar 29;15:10938. doi: 10.1038/s41598-025-91568-x (PMC11954960; doi:10.1038/s41598-025-91568-x)
Supplement: Supplementary file 2 — Supplementary Material 2 [file 41598_2025_91568_MOESM2_ESM.docx]

**Network Pharmacology and AI in Cancer Research Uncovering Biomarkers and Therapeutic Targets for RALGDS Mutations**

**S. Mohammed Zaidh^1,2^, Hariharan Thirumalai Vengateswaran^1^, Mohammad Habeeb^1^, Kiran Balasaheb Aher^3^, Girija Balasaheb Bhavar^4^, Irfan N^1*^,** **K.N.V. Chenchu Lakshmi^5^**

**Supplementary 2**

**Pathway Analysis genes involved**

The genes and elements involved in the pancreatic adenocarcinoma pathway from the provided image are KRAS, RALGDS, RALA, RALB, RAC1, CDC42, RAC2, RAC3, PLD1, PA, PIP3, PIK3CA, PIK3CB, PIK3CD, PIK3CG, PIK3R1, PIK3R2, PIK3R3, AKT1, AKT2, AKT3, BAD, CASP9, BCL2L1, CHUK, IKBKB, IKBKG, RELA, NFKB1, MTOR, RPS6KB1, RPS6KB2, VEGFA, CDKN2A, CDK4, CDK6, CCND1, RB1, E2F1, E2F2, E2F3, TP53, CDKN1A, CDKN1B, DDB2, BAK1, BAX, GADD45A, GADD45B, GADD45G, POLK, BRCA2, RAD51, MAP2K1, MAP2K2, MAPK1, MAPK3, MAPK6, MAPK9, MAPK10, RHOA, PAK3, PAK4, PAK6, PAK1, PAK2, PAK5, BUB1B-PAK6, RAC1, RAC2, RAC3, ARHGEF6, TIAM1, PRKCD, PRKCI, PEBP1, DUSP6, TGFA, EGF, ERBB2, EGFR, JAK1, STAT1, STAT3, TGFBR1, TGFBR2, SMAD2, SMAD3, SMAD4, TGF1, TGF2, TGF3, and RALBP1.

The genes and elements involved in the chromosomal and microsatellite instability in colorectal cancer pathway are TGFB1, TGFB2, TGFB3, TGFBR1, TGFBR2, SMAD2, SMAD3, SMAD4, BCL2, PMAIP1, BAD, BCL2L11, BBC3, CYCS, BAX, MSH6, MSH3, MSH2, MLH1, AKT1, AKT2, AKT3, PI3K, CASP9, KRAS, ARAF, RAF1, BRAF, MAP2K1, MAPK1, MAPK3, MAPK8, MAPK9, MAPK10, JUN, FOS, MYC, AXIN1, APC, AXIN2, APC2, GSK3B, CTNNB1, CSNK1A1, CSNK1A1L, CSNK1D, TCF7, TCF7L2, LEF1, TCF7L1, RALGDS, RALA, RALB, RAC1, RAC2, RAC3, RHOA, REL, EXOC2, TBK1, APPL1, CASP3, NTNT1, DCC, PTGS2, PGH2, PGE2, CDKN1A, DDB2, POLK, TP53, BIRC5, CCND1, MYC, and other related pathways and genes such as BCL2L11, TCF7L1, TCF7L2, LEF1, BAX, BAK1, JUN, FOS, MYC, REL, and MAPK8.

**Network Pharmacology**

The major genes are involved in alteration studies network pharmacology listed in the image are ARAF, CNKSR2, KRAS, BRAF, PIK3CA, CALM1, MAPK1, MLLT4, EGFR, MAP3K3, RALGDS, MAP3K2, PDE6D, RABHRAS, NRAS, SOS2, SOS1, LZTR1, SPRED1, NF1, PTPN11, RAF1, SHOC2, and MAP2K1.

1.3 **Docking**


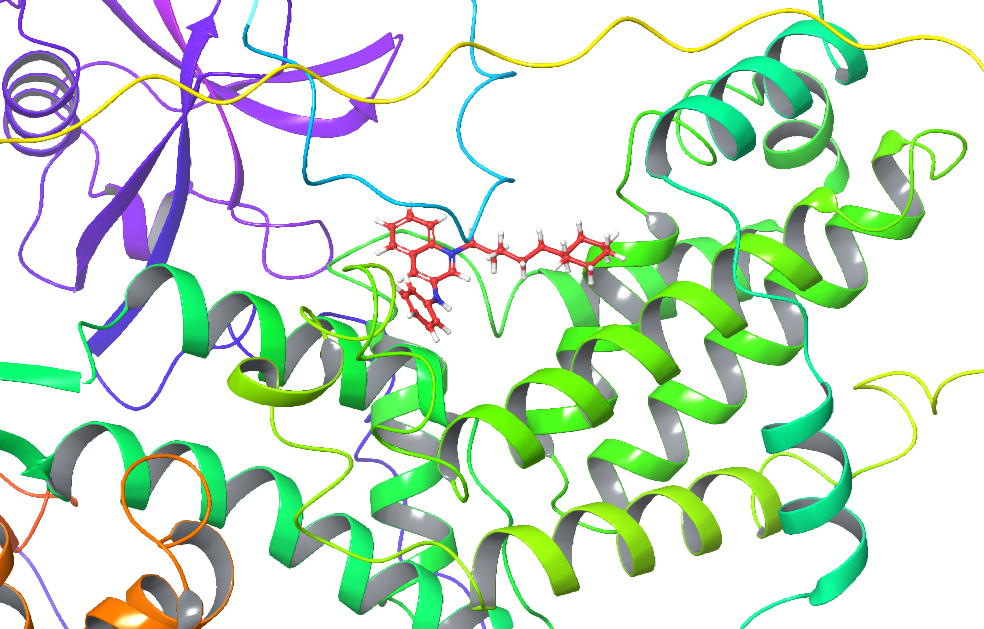


**Figure 1:** inside in the protein interaction Lead 1


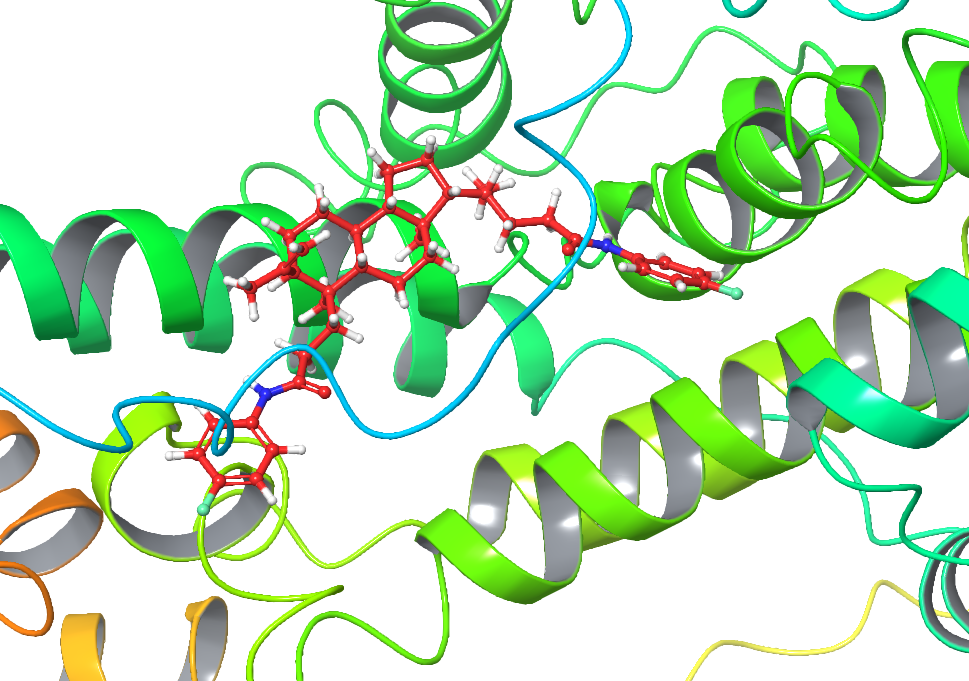


**Figure 2:** inside in the protein interaction Lead 2


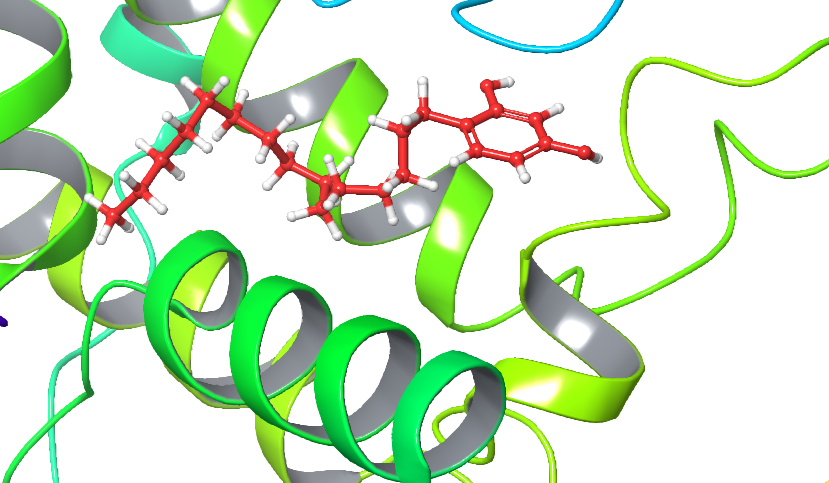


**Figure 2:** inside in the protein interaction Lead 3

**Retrosynthetic prediction of best lead molecule of octyl ring system**

The depicted reaction mechanism involves the coupling of an amine (Ar1-NH2) with a boronic acid (Ar2-B(OH)2). Initially, the nitrogen atom from the amine nucleophilically attacks the boron atom of the boronic acid, forming an intermediate complex. This intermediate then undergoes dehydration, eliminating water molecules and leading to the formation of a stable boron-nitrogen bond. The final product is an aryl-substituted amine (Ar1-NH-Ar2), where the nitrogen is bonded to both aromatic groups Ar1 and Ar2. This reaction typically proceeds under conditions that facilitate the dehydration step, such as elevated temperatures or the presence of a dehydrating agent illustrated in figure S3.


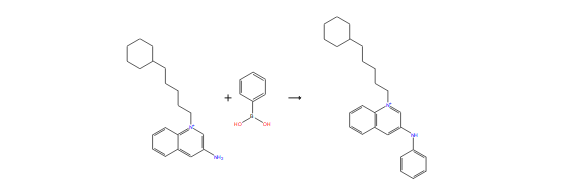


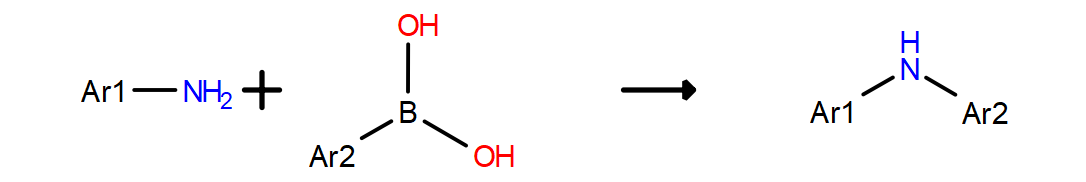


**Figure 3** Retrosynthetic pathway of Octyl ring system


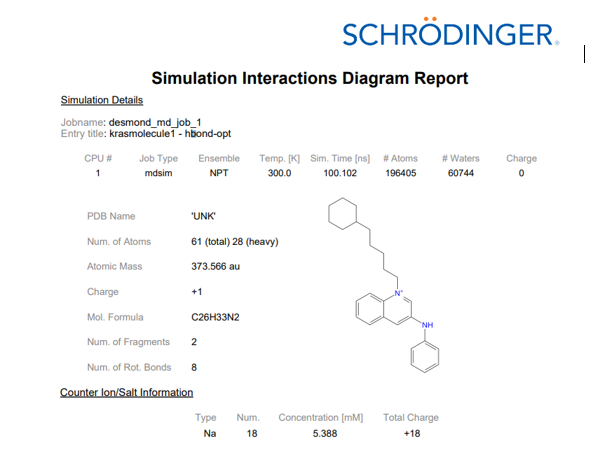


**Figure 4** simulation KRAS with lead 1


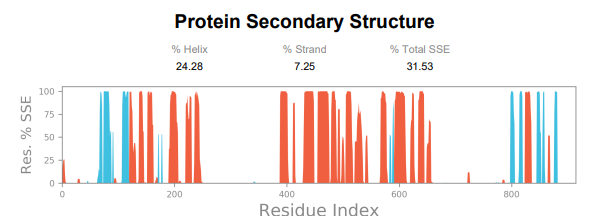
\

**Figure 5:** Secondary structure of protein.


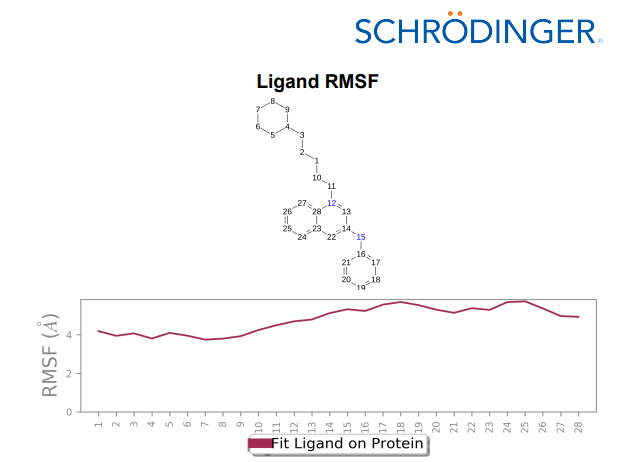


**Figure 6:** Ligand RMSF of the lead 1 Octyl ring system

The energy profiles in the Ligand Torsion Profile diagrams depict how the ligand's potential energy changes with the rotation around specific bonds. Each graph displays energy variations as a function of the torsion angle, identifying the most and least favorable conformations by their energy minima and maxima. The superimposed histograms show the frequency of each torsion angle, with peaks indicating the most energetically favorable conformations. This integrated analysis of energy profiles and torsion angle distributions provides valuable insights into the ligand's preferred conformations and stability, crucial for predicting its interactions in biological contexts. It was illustrated in figure S7.


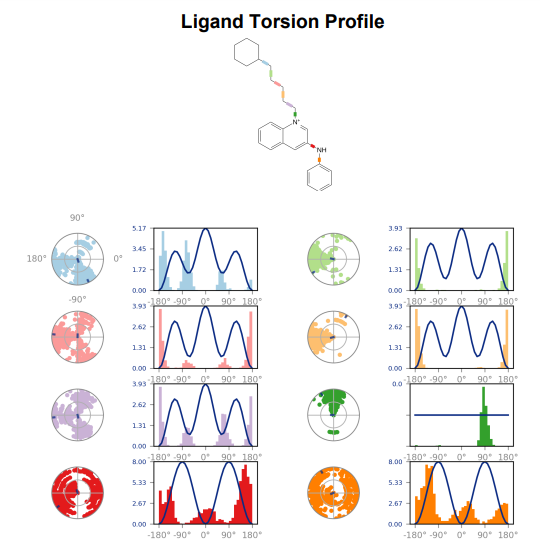


**Figure 7** lead1 Octyl rings torsion of the 100ns simulation.


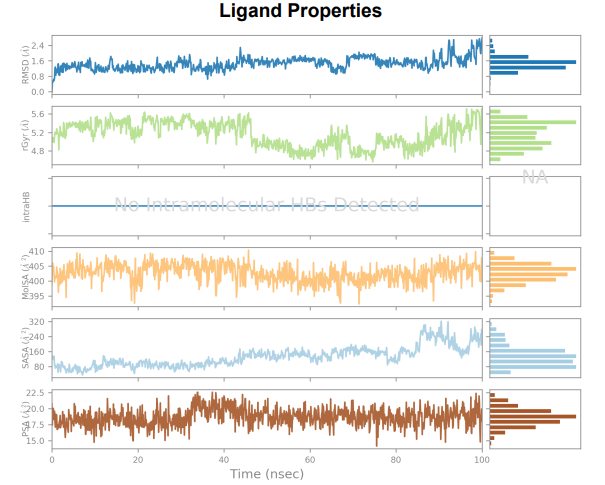


**Figure 8** Ligand properties lead 1 octyl rings system
